# Research on the Error of Global Positioning System Based on Time Series Analysis

**DOI:** 10.3390/s22103614

**Published:** 2022-05-10

**Authors:** Lijun Song, Lei Zhou, Peiyu Xu, Wanliang Zhao, Shaoliang Li, Zhe Li

**Affiliations:** 1 School of Information and Control Engineering, Xi’an University of Architecture and Technology, Xi’an 710055, China; zhoulei971231@xauat.edu.cn (L.Z.); xupeiyu155050@163.com (P.X.); xktiger@126.com (Z.L.); 2 Shanghai Aerospace Control Technology Institute, Shanghai 201100, China; zhaodada999@163.com (W.Z.); li_shaoliang@126.com (S.L.); 3 Shanghai Engineering Research Center of Inertia, Shanghai 201100, China

**Keywords:** Global Positioning System (GPS), Time Series Analysis (TAS), Autoregressive (AR) model, Kalman filtering, positioning precision

## Abstract

Due to the poor dynamic positioning precision of the Global Positioning System (GPS), Time Series Analysis (TSA) and Kalman filter technology are used to construct the positioning error of GPS. According to the statistical characteristics of the autocorrelation function and partial autocorrelation function of sample data, the Autoregressive (AR) model which is based on a Kalman filter is determined, and the error model of GPS is combined with a Kalman filter to eliminate the random error in GPS dynamic positioning data. The least square method is used for model parameter estimation and adaptability tests, and the experimental results show that the absolute value of the maximum error of longitude and latitude, the mean square error of longitude and latitude and average absolute error of longitude and latitude are all reduced, and the dynamic positioning precision after correction has been significantly improved.

## 1. Introductions

The United States was the first country to start the new generation of space satellite navigation and position system. Subsequently, Russia and China have studied global navigation positioning systems and developed them rapidly. The research and development of GPS was to serve military needs and, as early as 1972, the United States Navy transit project and the Air Force 621B project realized the application of GPS. With the development of economic globalization, smart phones, vehicles and ships, which are based on the combination of GPS and modern technology, have gradually penetrated into people’s daily life [1]. A country’s important national infrastructure needs a perfect and stable Global Positioning System, and the precision is an important parameter in the development of GPS [2].

At present, there are two methods to improve the positioning precision of GPS. The first is to use the Differential Global Positioning System (DGPS) [3]. However, this method has several limitations: (1) the equipment limitations: the receiver of a differential signal must be used in practical applications. (2) Restrictions of work area: the work area is limited by the differential network of a wide area. (3) Non-autonomous: the transmitting source is required to improve precision [4]. The other is to use the positioning data which are collected by the receiver for error correction. This method has poor real-time performance and is vulnerable to external interference [5,6]. Therefore, it is particularly important to improve the positioning precision of GPS without changing the hardware device.

Scholars have built the error model of GPS to improve the positioning precision of GPS. In Ref. [7], Zhao Shan et al. used the ARMA (3,2) model to obtain the single error model of a user by the combination of position operation and clock displacement filtering, which has a certain stability. In Ref. [8], Liu Di et al. made a long-term observation of a static point to obtain the same characteristics of error sequence and elevation error sequence between longitude and latitude, and finally established the AR (n) error model of GPS. In Ref. [9], Wang Rong et al. analyzed the error of GPS and established the error model of GPS. In Ref. [10], Zhiqiang Liu et al. modeled the time-selective channel as an AR process and used a Kalman filter to track the time change. In Ref. [11], Tan-Jan Ho et al. proposed a framework modeling which is based on multiple AR models, and developed the channel predictor MAR. In Ref. [12], Christos Komnanakis et al. used a Kalman filter to track a low-order autoregressive model, which is similar to the change in multiple input and multiple output channels.

The models used AR models based on time series by the scholars. If the output sequence of a model is $x_{t}$, there is a non-linear problem of parameter estimation, and the algorithm is complex and difficult. In order to solve the non-linear problems and effectively improve the positioning precision of GPS, we use the TAS and Kalman filter to analyze the characteristics of positioning error data, set up the Autoregressive (AR) model of GPS, complete the parameter estimation of the AR model by the least square method, and estimate the state of the system by a Kalman filter, so as to make the positioning precision more reliable and accurate [13].

## 2. The Error Model of GPS

The error data of GPS are a discrete random variable of time series, which is different from the analyzed dynamic data because the time series is a realization of random processes and has a different physical background [14,15]. The processing method of the corresponding model is an approximate description according to the data characteristics, to determine the type of model suitable for the time series [16].

There are three important models of finite order linear in TSA, Moving Average (MA) model, Autoregressive (AR) model, and Autoregressive Moving Average (ARMA) model [17]. The type of model can be determined by analyzing the autocorrelation and partial correlation of the error signal, and the identification methods of the three models are shown in Table 1.

As shown in Table 1, the autocorrelation function of MA is truncated, while the partial autocorrelation function is trailing. The autocorrelation function and partial autocorrelation function of ARMA are trailing. The autocorrelation function of AR has the trailing property, while the partial autocorrelation function has the truncation property, where truncation means that the time-order autocorrelation function or partial autocorrelation function is 0 when the order is greater than a constant K. The trailing property means that the autocorrelation function or partial autocorrelation function fluctuates near zero after a certain order.

If a time series is generated by a certain type of model, it should theoretically have corresponding statistical characteristics [18,19]. Therefore, the sample autocorrelation function and partial autocorrelation function of time series can be calculated, and the characteristics can be compared with the characteristics of a theoretical autocorrelation function and partial autocorrelation function of different types of series, and then the model type suitable for the series can be judged [20].

The estimated value ${\hat{\rho }}_{k}$ of the autocorrelation function is a measurement to describe the dependence between values of random processes at different times.

After obtaining the error observation data $X_{i}\left(i=1,2,\cdots ,N\right)$, the sample autocorrelation function ${\hat{\rho }}_{k}$ of the error sequence is

(1) $${\hat{\rho }}_{k}={\hat{\gamma }}_{k}/\gamma_{0}$$

where the estimate value of autocovariance is ${\hat{\gamma }}_{k}=\frac{1}{N}\sum_{i=1}^{N-k}\left(X_{i}-X_{M}\right)\left(X_{i+k}-X_{M}\right)$, k=0,1,⋯. The estimate data of mathematical expectation are $X_{M}$.

Using a numerical method and MATLAB programming, the response simulations of the autocorrelation function and partial autocorrelation function of longitude and latitude are obtained.

**Figure 1:**
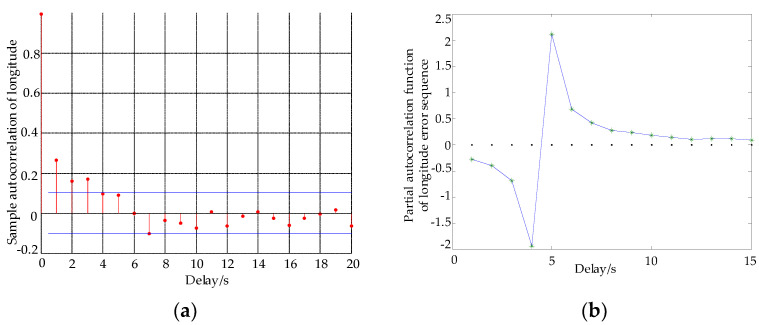
Autocorrelation and partial autocorrelation of longitude. (**a**) Longitude autocorrelation function, (**b**) longitude partial autocorrelation function.

**Figure 2:**
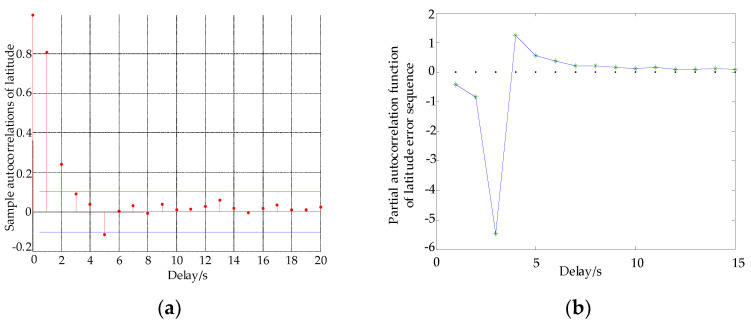
Autocorrelation and partial autocorrelation of latitude. (**a**) Latitude autocorrelation function, (**b**) latitude partial autocorrelation function.

By using ${\hat{\gamma }}_{k}$ instead of $\gamma_{k}$, the estimation of the partial autocorrelation function can be obtained recursively. If the autocorrelation function ${\hat{\varphi }}_{kk}$ of the sample is truncated in step, it can be determined as an AR (M) sequence. If ${\hat{\varphi }}_{kk}$ is not truncated, it is an ARMA sequence. From Figure 1a and Figure 2a, the error autocorrelation sequences of longitude and latitude have coordinates fluctuations near zero after order 4, the red line represents the autocorrelation function data of latitude and longitude error. From Figure 1b and Figure 2b, the autocorrelation function of longitude and latitude shows trailing, and the partial autocorrelation function shows truncation, the blue line represents the partial correlation function error data of latitude and longitude error, * represents the data point of latitude and longitude error per second. Therefore, the error of longitude and latitude can be expressed by the AR (M) model, that is,

(2) $$\{\begin{array}{c} {\hat{\varphi }}_{11}={\hat{\gamma }}_{1}/{\hat{\gamma }}_{0}\\ {\hat{\varphi }}_{\begin{array}{cc} k+1 & k+1 \end{array}}=\left({\hat{\gamma }}_{k+1}-\sum_{i=1}^{k}{\hat{\varphi }}_{kj}{\hat{\gamma }}_{k+1-j}\right)/\left({\hat{\gamma }}_{0}-\sum_{i=1}^{k}{\hat{\varphi }}_{kj}{\hat{\gamma }}_{j}\right)\\ {\hat{\varphi }}_{\begin{array}{cc} k+1 & j \end{array}}={\hat{\varphi }}_{kj}-{\hat{\varphi }}_{\begin{array}{cc} k+1 & k+1 \end{array}}{\hat{\varphi }}_{\begin{array}{cc} k & k+1-j \end{array}},j=1,2,\cdots ,k \end{array}.$$

The recursive formula of ${\hat{\rho }}_{k}$ can be used to find ${\hat{\varphi }}_{kk}$ by using ${\hat{\rho }}_{k}$ instead of ${\hat{\gamma }}_{k}$.

## 3. The Establishment of the Error Model of GPS

### 3.1. Preamble Data Processing

**Figure 3:**
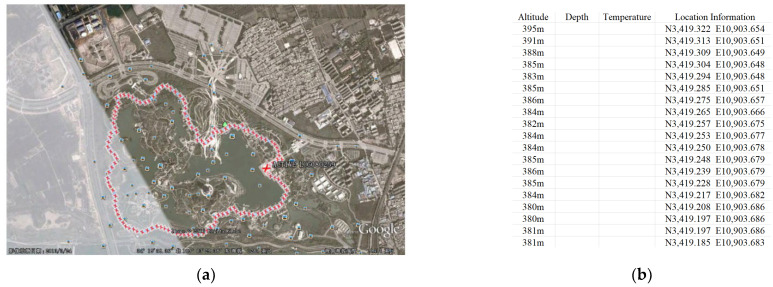
Google Earth motion track map longitude and latitude information. (**a**) Google Earth motion map. (**b**) Longitude and latitude coordinate information.

We use Google Earth, an interactive electronic map, to capture longitude and latitude information and motion track. From Figure 3a,b, Figure 3a is the Google Earth motion map, a red cross is the trajectory point and a square is an ordinary landmark. Figure 3b is partial latitude and longitude data.

The longitude and latitude data are established in the WGS-84 coordinate system, and the unit is degree-minute second. However, the data in the unit are required, and coordinate projection is required [21]. The WGS-84 coordinate value is projected into the rectangular coordinate value of the Gaussian projection plane by ARCGIS software [22]. As shown in Table 2, the X coordinate is north latitude and the Y coordinate is east longitude.

Due to the randomness of the point position in the dynamic environment, the truth value of the observed quantity cannot be obtained directly, which makes the error separation relatively difficult [23]. In order to obtain the position data in the dynamic environment, we adopt the polynomial fitting method to separate the error and select the power series fitting, and the fitting value is the true value of the motion trajectory [24]. According to the polynomial approximation theory, the fitting value compares with the position data continuously collected on this motion trajectory to obtain the estimation of the GPS measurement error value, so as to separate the error data of GPS in the dynamic environment.

The estimated value of GPS measurement error is as follows:

(3) $${\hat{X}}_{GPS}=x_{i}-f\left(b,t\right)$$

where $x_{i}$ is the measured value. $f\left(b,t\right)=\sum_{i=1}^{n+1}b_{i}t^{n+1-i}$ is an n-order power function fitting polynomial model. Coefficient $b_{i}$ is a $\chi^{2}$ solution with minimum quantity, $\chi^{2}\left(b\right)=\overset{N}{\underset{i=1}{\sum }}{\left(\frac{x_{i}-f\left(b,t_{i}\right)}{{\Delta x}_{i}}\right)}^{2}$, ${\Delta x}_{i}$ is the deviation between the original data and the fitted value. If the order of the model polynomial is too low, the fitting is rough. If the order is too high, overfitting will make the model contain data noise. In practical applications, it is necessary to judge whether the fitting is appropriate by the value of fitting factor Q. If Q is close to 0.5, it indicates that the fitting is good.

(4) $$Q\left(\chi^{2},N-n-1\right)=1-P(\chi^{2}<\left(N-n-1\right))$$

where P is the probability, N is the number of samples, and n is the order of the model.

**Figure 4:**
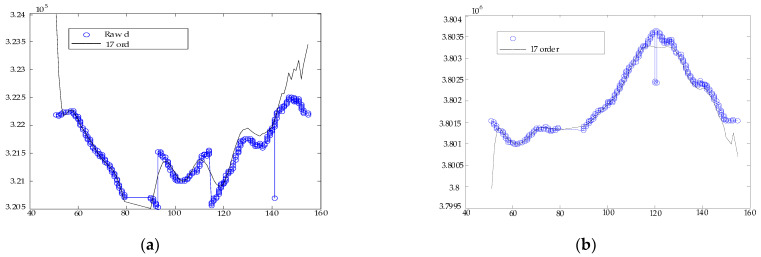
Comparison of measured data and fitted data of GPS. (**a**) The measured data and fitted data of longitude. (**b**) The measured data and fitted data of latitude.

**Figure 5:**
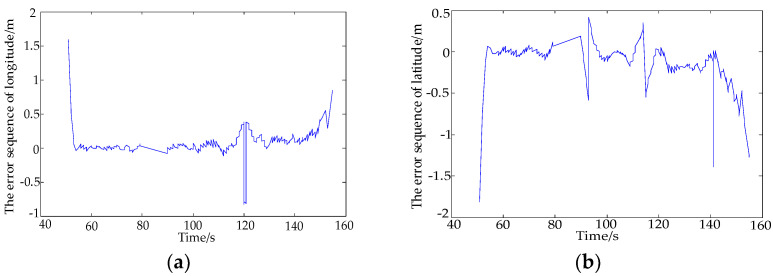
The error sequence of GPS. (**a**) The error sequence of longitude. (**b**) The error sequence of latitude.

The calculated Q value is 0.408 after fitting. In Figure 4, there is the large fluctuation of some points which is caused by large-scale movement, not a bad value. In Figure 5, it is the result of error separation of the dynamic position data. Taking the fitting value as the true value estimation of the motion trajectory, and comparing it with the data of position, the error value of dynamic measurements can be obtained by the motion trajectory.

### 3.2. Determination and Parameter Estimation of AR Model Order

It is known that the order of the AR (M) model is the determination of the AR model order when the probability of the partial autocorrelation function falling within the interval $\left|\frac{2}{\sqrt{n}}\right|$ exceeds 95% [25]. We take $\frac{2}{\sqrt{n}}=\frac{2}{\sqrt{329}}=0.11$ and obtain Table 3 and Table 4 by a MATLAB programming calculation, and the partial autocorrelation functions of longitude and latitude are truncated in 10 steps. The error model of GPS can be expressed as AR (10), that is,

(5) $$y\left(k\right)=\sum_{i=1}^{10}\varphi_{i}y\left(k-i\right)+b\left(k\right)$$

where $\varphi_{i}$ is an autoregressive coefficient, b(k) is the white noise with mean value of 0 and variance of $\theta^{2}$.

As the least square estimation is a precise estimation of model parameters, the estimation precision is high [26]. Therefore, the least square method is used to estimate parameters. It is

(6) $${\hat{\varphi }}_{L}={\left(X^{T}X\right)}^{-1}X^{T}Y.$$

The variance of b(k) is:

(7) $$\theta^{2}=\frac{1}{I-n}\sum_{t=n+1}^{I}\leq \left(y\left(k\right)-\varphi_{1}y\left(k-1\right)-\cdots -\varphi_{i}y\left(k^{2}-n\right)\right).$$

The autoregressive coefficient and noise variance obtained by the least square method are shown in Table 4.

### 3.3. Applicability Test of AR Model

The applicability of the model is essentially to test whether it is a white noise sequence, and the most important problem is to test the independence of the sequence [27]. When the number of samples is enough, the autocorrelation functions of residual error are uncorrelated and approximate to the normal distribution.

(8) $${\hat{\rho }}_{k}=\sum_{i=1}^{i-k}{\hat{b}}_{1}{\hat{b}}_{1+k}/\left[\sum_{t-1}^{i}{\hat{b}}^{2}\right].$$

At the significance level α=0.05, $\left|{\hat{\rho }}_{k}\right|\leq \frac{1.96}{N^{0.5}}$, it is acceptable to assume that ${\hat{\rho }}_{k}=0$ is independent.

**Figure 6:**
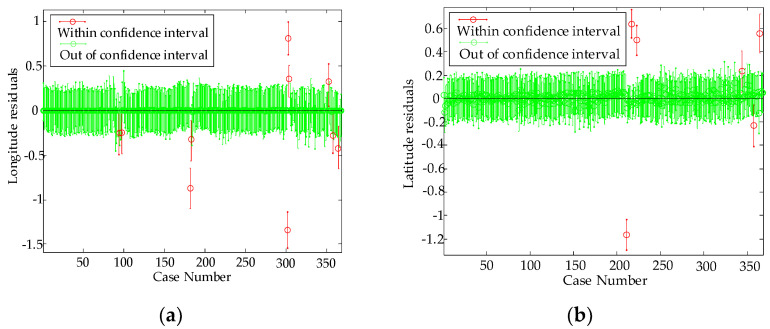
Autocorrelation function of residual sequence. (**a**) Autocorrelation function of longitude residual sequence. (**b**) Autocorrelation function of latitude residual sequence.

The green symbol is the residual value within the confidence interval, and the red symbol is the residual value outside the confidence interval. The steadiness of the model is also an indicator to test the residual sequence. It can be seen from Figure 6 that the autocorrelation coefficient of residual sequence fluctuates randomly with 0 as the mean in the 95% confidence boundary, and the residual sequence data of the longitude and latitude are stable, which indicates that the model has a high degree of fitting with the actual system and the model meets the requirements. Therefore, the AR (10) model is more suitable for GPS longitude and latitude error time series.

## 4. The Application of Kalman Filter in the Error Model

### 4.1. Discretization of State Equations of Continuous Systems

The actual physical system is generally continuous, and the dynamic characteristics are described by continuous differential equations. Therefore, the discretizations of the system equation and the observation equation are needed [28,29].

The system state equation describing the dynamic characteristics of the physical system is:

(9) $$\dot{X}(t)=f(t)X(t)+g(t)v(t)$$

where the driving source v(t) of the system is the white noise process, which is

(10) $$\{\begin{array}{c} E[v(t)]=0\\ E[v(t)v^{T}(\tau )]=q\sigma (t-\tau ) \end{array}$$

where q is v(t) variance intensity matrix. *σ*(t-*τ*) is a function of Dirac *σ*.

According to the linear system theory, the discretization of the system state equation is:

(11) $$X(t_{k+1})=a(t_{k+1},t_{k})X(t_{k})+\int_{t_{k}}^{t_{k+1}}a(t_{k+1},\tau )g(\tau )v(\tau )d\tau$$

where the one-step transfer matrix a ${(t}_{k+1}{,t}_{k})$ satisfies the equation:

(12) $$a_{k+1,k}=a(t_{k+1},t_{k})=I+Tf_{k}+\frac{T^{2}}{2!}f_{k}^{2}+\frac{T^{3}}{3!}f_{k}^{3}+\frac{T^{4}}{4!}f_{k}^{4}+\cdots$$

where $f_{k}{=f(t}_{k}{),T=t}_{k+1}{-t}_{k}$. The equation is the real-time calculation formula of a one-step transfer matrix.

The discretization state equation of continuous system also includes the equivalent discretization of the excited white noise process v(t).

(13) $$V_{k}=\int_{t_{k}}^{t_{k+1}}\phi (t_{k+1},\tau )g(\tau )v(\tau )d\tau .$$

Equation (11) can be abbreviated as:

(14) $$X_{k+1}=a_{k+1,k}X_{k}+V_{k}$$

where $X_{k}{=X(t}_{k})$. Then, for V(k) defined in Equation (13), it is:

(15) $$\{\begin{array}{c} E[V_{k}]=0\\ E[V_{k}V_{j}^{T}]=\int_{t_{k}}^{t_{k+1}}\phi (t_{k+1},t_{k})g(t)qg^{T}(t)\phi^{T}(t_{k+1},t_{k})dt=Q\delta_{kj} \end{array}$$

where, $\delta_{\mathrm{kj}}$ is Kronecker δ function. The variance matrix $Q_{k}$ of $V_{k}$ satisfies the following equation:

(16) $$M_{1}=g_{k}qg_{k}{}^{T}$$

(17) $$M_{i+1}=f_{k}qg_{k}{}^{T}$$

(18) $$Q_{k}=TM_{1}+\frac{T^{2}}{2!}M_{2}+\frac{T^{3}}{3!}M_{3}+\frac{T^{4}}{4!}M_{4}+\cdots$$

where, $g_{k}{=g(t}_{k})$, the Equation (16) is the real-time calculation formula of $Q_{k}$.

### 4.2. The Basic Equation of Discrete Kalman Filter

Kalman is linear minimum variance estimation [30]. For Kalman model, the state equation and observation equation of discrete linear system are respectively:

(19) $$X_{k}=a_{k,k-1}X_{k-1}+\xi_{k-1}V_{k-1}$$

(20) $$Y_{k}=c_{k}X_{k}+W_{k}$$

where, $a_{k,k-1}$ is one step transition matrix of time, which is $t_{k-1}$ to $t_{k}$. $c_{k}$ is the measurement matrix. $\xi_{k-1}$ is the system noise driving. $W_{k}$ is the observation noise sequence. $V_{k-1}$ is the excitation noise sequence of the system. Both $V_{k}$ and $W_{k}$ are satisfied

(21) $$\{\begin{array}{c} {E(V}_{K}{)=0,E(V}_{K}V_{j}^{T}{)=0,=Q}_{K}\delta_{\mathrm{Kj}}\\ {E(W}_{K}{)=0,E(W}_{K}W_{j}^{T}{)=0,=R}_{K}\delta_{\mathrm{Kj}}\\ {E(W}_{K}V_{j}^{T})=0 \end{array}$$

where, $Q_{k}$ is the variance matrix of the system noise sequence, it is a non-negative matrix. $R_{k}$ is the variance matrix of noise sequence on both sides, it is positive definite matrix. $\delta_{\mathrm{kj}}$ is Kronecker δ function.

If $X_{k}$ is conformed to Equation (19), the measured value $Y_{k}$ is conformed to Equation (20), the system noise $V_{k}$ and the measurement noise $W_{k}$ are conformed to Equation (21), and the system noise variance matrix $Q_{k}$ is non-negative definite, the measurement noise variance matrix $R_{K}$ is positive definite, and the measurement of time K is $Y_{k}$, the equation can be solved for $X_{K}$ estimation ${\hat{X}}_{K}$.

Then

(22) $$\{\begin{array}{c} X_{k|k-1}=a_{k,k-1}{\hat{X}}_{k}\;\\ P_{k|k-1}=a_{k,k-1}P_{k-1}a^{T}{}_{k,k-1}+\xi_{k-1}Q_{k-1}\xi_{k-1}^{T}\;\\ K_{k}=P_{k|k-1}c_{k}^{T}{\left(c_{k}P_{k|k-1}c_{k}^{T}+R_{k}\right)}^{-1}\\ {\hat{X}}_{k}={\hat{X}}_{k|k-1}+K_{k}\left(Y_{k}-c_{k}{\hat{X}}_{k|k-1}\right)\\ p_{k}=\left(I-K_{k}c_{k}\right)P_{k|k-1}{\left(I-K_{k}c_{k}\right)}^{T}+K_{k}R_{k}K_{k}{}^{T} \end{array}$$

where, $X_{k|k-1}$ is the one-step prediction equation of state. $P_{k|k-1}$ is the one-step prediction of mean square error. $K_{k}$ is the gain equation of filter. ${\hat{X}}_{k}\;$ is the estimation equation of filter. $p_{k}\;$ is the optimal mean square error at time K.

As long as the initial value ${\hat{X}}_{0}$ and $P_{0}$ is given, according to the measurement $Y_{k}$ at time k, the estimated state ${\hat{X}}_{K}(k=1,2,3,\cdots )$ at time k can be deduced.

### 4.3. Kalman Filter Based on AR Model

The state vector of GPS is

(23) $$x\left(k-1\right)=\left[\begin{array}{c} x\left(k-10\right)\\ x\left(k-9\right)\\ \vdots \\ x\left(k-1\right) \end{array}\right]$$

The model is based on $x\left(k\right)=\sum_{n=1}^{10}\varphi_{n}x\left(k-n\right)+b\left(k\right)$, the state space model of Kalman filter model is Equations (9) and (10), where $V_{k}$ and $W_{k}$ have statistical characteristics of Equation (21).

The process noise turn the difference equation of AR (10) model into state Equation (24), it is

(24) $$\left[\begin{array}{c} x\left(k-9\right)\\ x\left(k-8\right)\\ \vdots \\ x\left(k\right) \end{array}\right]=\left[\begin{array}{ccccc} 0 & 1 & 0 & \cdots & 0\\ 0 & 0 & 1 & 0 & 0\\ \vdots & \vdots & & & \vdots \\ 0 & 0 & \cdots & \cdots & 1\\ \varphi_{10} & \varphi_{9} & \cdots & \cdots & \varphi_{1} \end{array}\right]\left[\begin{array}{c} x\left(k-10\right)\\ x\left(k-9\right)\\ \vdots \\ x\left(k-1\right) \end{array}\right]+\left[\begin{array}{c} 0\\ 0\\ \vdots \\ 0\\ 1 \end{array}\right]V\left(k\right)$$

Let $\left[\begin{array}{ccccc} 0 & 1 & 0 & \cdots & 0\\ 0 & 0 & 1 & 0 & 0\\ \vdots & \vdots & & & \vdots \\ 0 & 0 & \cdots & \cdots & 1\\ \varphi_{10} & \varphi_{9} & \cdots & \cdots & \varphi_{1} \end{array}\right]=A,\left[\begin{array}{c} 0\\ 0\\ \vdots \\ 0\\ 1 \end{array}\right]=B$, Equation (24) can also be abbreviated as

(25) x(k)=Ax(k−1)+BV(k)

The observation equation is

(26) y(k)=x(k)+W(k)=Cx(k)+W(k)

where, $C=\left[\begin{array}{ccccc} 0 & 0 & \cdots & 0 & 1 \end{array}\right]$.

Because the statistical characteristics of V(k) and W(k) are consistent with Kalman filter, the recursive expression of Kalman filter based on AR model is show as:

(27) $$\{\begin{array}{c} {\hat{X}}_{k|k-1}=A{\hat{X}}_{k-1}\\ P_{k|k-1}=AP_{k-1}A^{T}+BQ_{k-1}B^{T}\\ K_{k}=P_{k|k-1}c^{T}{\left(cP_{k|k-1}c^{T}+R\right)}^{-1}\\ {\hat{X}}_{k}={\hat{X}}_{k|k-1}+K_{k}\left(Y_{k}-c_{k}{\hat{X}}_{k|k-1}\right)\\ P_{k}=\left(I-K_{k}c_{k}\right)P_{k|k-1} \end{array}$$

where, $X_{k|k-1}\;$ is the one-step prediction equation of filter state. $P_{k|k-1}$ is the one-step prediction of mean square error. $K_{k}$ is the gain equation of filter. If the Kalman filter gain value $K_{k}$ is very small, the filtering result is closer to the recursive result of the system state estimation value. If $K_{k}$ is large, the filtering result is closer to the state variable calculated of the observed value. ${\hat{X}}_{k}\;$ is the estimation equation of filter. $p_{k}\;$ is the optimal mean square error at time K.

There are two ways to deal with Q: one is that Q is a certain value. The second is that Q is an uncertain random variable. Therefore, the Q value in this paper is a definite value, which is the process noise variance of the system, and its value is $\sigma *I_{10\mathrm{order}}$. When the state transition process has been determined, the smaller of Q is the better. When Q gradually increases, the convergence of filter slows down and the disturbance of the state variable becomes larger. The value of R is related to the characteristics of the device and is the input value of the filter.

## 5. Simulation and Analysis

### 5.1. AR (10) Model Kalman Filter Experiment

**Figure 7:**
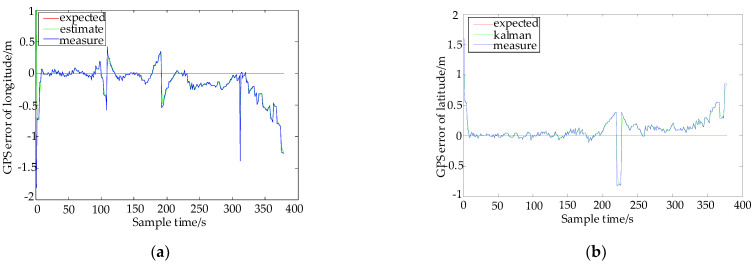
The error filtering results of longitude and latitude. (**a**) The error of longitude. (**b**) The error of latitude.

The dynamic error model (AR model) of GPS uses a Kalman filter, Q/R is 0.1/0.5, and the order of the AR model is determined to be 10. The error filtering results of longitude and latitude are shown in Figure 7.

In Figure 7, the blue curve is the error data of original longitude and latitude, the green curve is the error of longitude and latitude after the Kalman filter, and the red curve is the ideal error. The blue and green lines do not completely coincide, and the green lines after the Kalman filter are smoother. There are several points with large amplitude in the figure, which are caused by excessive movement when collecting data. In order to analyze Figure 7, Table 5 is shown below for explanation.

The analysis of data is carried out, and the absolute value of maximum error, mean square error, and mean absolute error are selected to reflect the changes in data before and after the filtering model. It can be seen from Table 5 that the absolute value of the maximum error of longitude and latitude after filtering is reduced by 30.50% and 37.26%. The mean square errors of longitude and latitude were reduced by 8.878% and 10.12%. The mean absolute errors of longitude and latitude were also reduced by 5.695% and 5.336%.

### 5.2. Compared Experiment of AR Model

**Figure 8:**
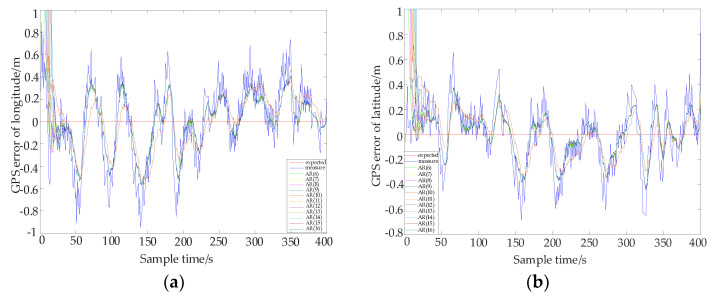
The longitude and latitude error curve of each model. (**a**) The error of longitude. (**b**) The error of latitude.

In order to further prove the effectiveness of the model in this paper, a new set of GPS data is collected, and an AR model based on a different order Kalman filter is established and compared with the model. Q/R is 0.1/0.5, the longitude and latitude error curve of each model is shown in Figure 8.

In Figure 8, the red line is the error of expectation, the blue line is the error of measurement, and the brown line the error of the AR (10) model. In order to more intuitively compare the error of each model, the longitude and latitude error data of each model are given in Table 6 and Table 7.

It can be seen from Table 6 and Table 7 that the absolute value of the maximum error of longitude and latitude in this paper has been reduced by 41.67% and 52.25%, respectively. The mean square errors of longitude and latitude were reduced by 37.41% and 39.48%, respectively. The mean absolute errors of longitude and latitude were also reduced by 36.53% and 23.26%, respectively. Although the errors of other order models are also greatly reduced with a Kalman filter, the AR (10) model in this paper is selected after order determination and adaptability tests, so the positioning accuracy of this model is better than other models.

## 6. Conclusions

In this study, the error of GPS is researched, and the statistical characteristics of GPS are analyzed and simulated. The least square method is applied to estimate the parameters, and obtain the basic equation of a discrete Kalman filter by the continuous Kalman filter. To eliminate the random error of GPS dynamic positioning data, the error model of GPS is combined with a Kalman filter and the experimental results show that the smaller the mean square error, the better the precision of the prediction model on the experimental data. The dynamic positioning precision after correction has been significantly improved, and this method can effectively improve the positioning precision of GPS and support for the application of GPS.

## Figures and Tables

**Table 1:** The correlation function characteristics of the model.

Model	Autocorrelation Function	Partial Autocorrelation Function
MA	Truncation	Trailing
AR	Trailing	Truncation
ARMA	Trailing	Trailing

**Table 2:** Rectangular coordinates of a Gaussian projection plane.

Point Number	1	2	3	4	5	6
X-coordinates	322,186.148	322,185.5618	322,159.4055	322,159.4055	322,158.8192	322,183.8031
Y-coordinates	3,801,532.042	3,801,501.221	3,801,470.887	3,801,470.887	3,801,440.067	3,801,408.76
Heights	395	391	388	385	383	385
Point number	7	8	9	10	11	12
X-coordinates	322,183.2169	322,208.2011	322,233.1854	322,233.1854	322,233.1854	322,232.5994
Y-coordinates	3,801,377.94	3,801,346.633	3,801,315.327	3,801,315.327	3,801,315.327	3,801,284.506
Heights	386	384	382	384	384	385
Point number	13	14	15	16	17	18
X-coordinates	322,232.0134	322,231.4273	322,256.4121	322,255.8262	322,255.2402	322,255.2402
Y-coordinates	3,801,253.686	3,801,222.866	3,801,191.559	3,801,160.739	3,801,129.918	3,801,129.918
Heights	386	385	384	381	380	380
Point number	19	20	21	22	23	24
X-coordinates	322,254.6543	322,254.6543	322,228.4972	322,202.34	322,480.8022	322,480.8036
Y-coordinates	3,801,099.098	3,801,099.098	3,801,068.764	3,801,038.43	3,801,022.048	3,801,022.039
Heights	381	381	384	386	384	385

**Table 3:** Partial correlation function of longitude and latitude.

Order Number	Partial Correlation Function of Longitude	Partial Correlation Function of Latitude
1	−0.2150	−0.2805
2	−0.7232	−0.4044
3	−2.5410	0.6886
4	1.5189	−1.9369
5	0.5226	2.1126
6	0.6765	0.6765
7	0.4181	0.4181
8	0.2553	0.2553
9	0.1881	0.1745
10	0.1089	0.1090
11	0.1063	0.1012
12	−0.1034	0.0989
13	−0.0217	0.0976
14	0.0344	0.09675
15	0.10384	0.09243
16	−0.0531	0.0881

**Table 4:** The data of autoregressive coefficient and noise variance.

	Longitude		Latitude	
Order Number	$\mathrm{Autoregressive}\;\mathrm{Coefficient}\varphi_{i}$	$\mathrm{Noise}\;\mathrm{Variance}\;\theta^{2}$	$\mathrm{Autoregressive}\;\mathrm{Coefficient}\varphi_{i}$	$\mathrm{Noise}\;\mathrm{Variance}\;\theta^{2}$
1	0.5750	6.07993 × 10^−6^	0.9146	8.0237 × 10^−6^
2	0.2136		0.0211	
3	0.0846		0.0104	
4	0.0722		0.0123	
5	−0.0485		−0.0035	
6	0.0074		−0.4411	
7	0.0276		0.3617	
8	0.0030		0.0561	
9	0.0661		0.0032	
10	0.0127		0.0064	

**Table 5:** The original error sequence and filtered error sequence of longitude and latitude.

	Absolute Value of Maximum Error/m	Mean Square Error/m	Mean Absolute Error/m
Longitude original error sequence	1.8125	0.266574	0.179414
Longitude filtered error sequence	1.2597	0.242909	0.169197
Latitude original error sequence	1.594	0.206185	0.123304
Latitude filtered error sequence	1	0.185163	0.116725

**Table 6:** The longitude errors of different models.

	Absolute Value of Maximum Error/m	Mean Square Error/m	Mean Absolute Error/m
Original error	0.9474	0.209081	0.278591
AR (6)	0.5683	0.137044	0.177516
AR (7)	0.5633	0.137429	0.177733
AR (8)	0.5584	0.137194	0.177533
AR (9)	0.5614	0.137537	0.178879
AR (10)	0.5526	0.130864	0.176819
AR (11)	0.5605	0.137209	0.180477
AR (12)	0.5692	0.135520	0.179191
AR (13)	0.5528	0.135469	0.191706
AR (14)	0.5597	0.135521	0.177114
AR (15)	0.5628	0.137509	0.182409
AR (16)	0.5658	0.138854	0.186109

**Table 7:** The latitude errors of different models.

	Absolute Value of Maximum Error/m	Mean Square Error/m	Mean Absolute Error/m
Original error	0.9248	0.209406	0.270833
AR (6)	0.4301	0.127979	0.213698
AR (7)	0.4346	0.128573	0.212659
AR (8)	0.4379	0.129551	0.211016
AR (9)	0.4392	0.130159	0.211360
AR (10)	0.4258	0.126727	0.207835
AR (11)	0.4395	0.131173	0.209522
AR (12)	0.4322	0.128886	0.210099
AR (13)	0.4256	0.127212	0.209449
AR (14)	0.4416	0.127643	0.210880
AR (15)	0.4360	0.130439	0.212706
AR (16)	0.4546	0.133593	0.214826

## Data Availability

The raw/processed data required to reproduce these findings cannot be shared at this time as the data also form part of an ongoing study.
